# Incidence and risk of infections associated with EGFR-TKIs in advanced non-small-cell lung cancer: a systematic review and meta-analysis of randomized controlled trials

**DOI:** 10.18632/oncotarget.14707

**Published:** 2017-01-17

**Authors:** Yingtian Wang, Mingzhen Wang, Qiaoxia Wang, Zhiying Geng, Mingxiang Sun

**Affiliations:** ^1^ Department of Respiratory Medicine, Beijing Airport Hospital, Shunyi districts, Beijing, China; ^2^ Department of Respiratory Medicine, Dongying People’s Hospital, Dongying, Shandong, Shandong, China

**Keywords:** erlotinib, gefitinib, EGFR-TKIs, infections, non-small-cell lung cancer

## Abstract

Currently, the overall incidence and risk of infections with epidermal growth factor receptor (EGFR)-tyrosine kinase inhibitors (TKIs) in non-small-cell lung cancer (NSCLC) patients remained undetermined. We searched Pubmed for related articles published from 1 January 1990 to 31 November 2015. Eligible studies included prospective randomized controlled trials (RCTs) evaluating therapy with or without EGFR-TKIs in patients with NSCLC. Data on infections were extracted. Pooled incidence, Peto odds ratio (Peto OR), and 95% confidence intervals (CIs) were calculated. A total of 17,420 patients from 25 RCTs were included. The use of EGFR-TKIs significantly increased the risk of developing all-grade infections (Peto OR 1.48, 95%CI: 1.12-1.96, p = 0.006) in NSCLC patients, but not for severe (Peto OR 1.26, 95%CI: 0.96-1.67, p = 0.098) and fatal infections (Peto OR 0.81, 95%CI: 0.43-1.53, p = 0.52). Meta-regression indicated the risk of infections tended to increase with the treatment duration of EGFR-TKIs. No publication of bias was detected. In conclusion, the use of EGFR-TKIs significantly increased the risk of developing all-grade infectious events in NSCLC patients, but not for severe and fatal infections. Clinicians should be aware of the risks of infections with the administration of these drugs in these patients.

## INTRODUCTION

Lung cancer is one of the most common malignancies and the most frequent cause of cancer-related mortality worldwide [1]. Despite the significant improvement in chemotherapy regimen for the treatment of advanced non-small-cell lung cancer (NSCLC), the 5-year survival for these patients remains relative poor [2, 3]. Thus, novel agents are urgently needed to improve the prognosis of these patients.

The epidermal growth factor receptor (EGFR) is a member of the HER family of receptor tyrosine kinases which plays a critical role in regulating the development and progression of many solid tumors including NSCLC [4–6]. Thus, EGFR and its related signal pathway have been regarded as attractive therapeutic targets in the treatment of NSCLC [7, 8]. Currently, three anti-EGFR agents, gefitinib, erlotinib and afatinib have been approved for use in EGFR mutation-positive NSCLC patients [9, 10]. Although EGFR-TKIs are generally well tolerated, a pattern of adverse events such as skin rash, diarrhea, thromboembolic events and interstitial lung disease have been reported [11–15], which is different from traditional cytotoxic agents. Infections have been reported with anti-EGFR agents. In 2014, Qi et al. performed a meta-analysis and found a significantly increased risk of severe infectious events associated with the use of anti-EGFR mono-clonal antibodies cetuximab and panitumumab in cancer patients (RR 1.34, p = 0.003) [16]. However, whether EGFR-TKIs increase the risk of infections in NSCLC remains unknown. We thus perform this meta-analysis and systematic review of available randomized controlled trials to determine the overall incidence and risk of infections in NSCLC patients treated with these drugs.

## RESULTS

### Search results

**Figure 1:**
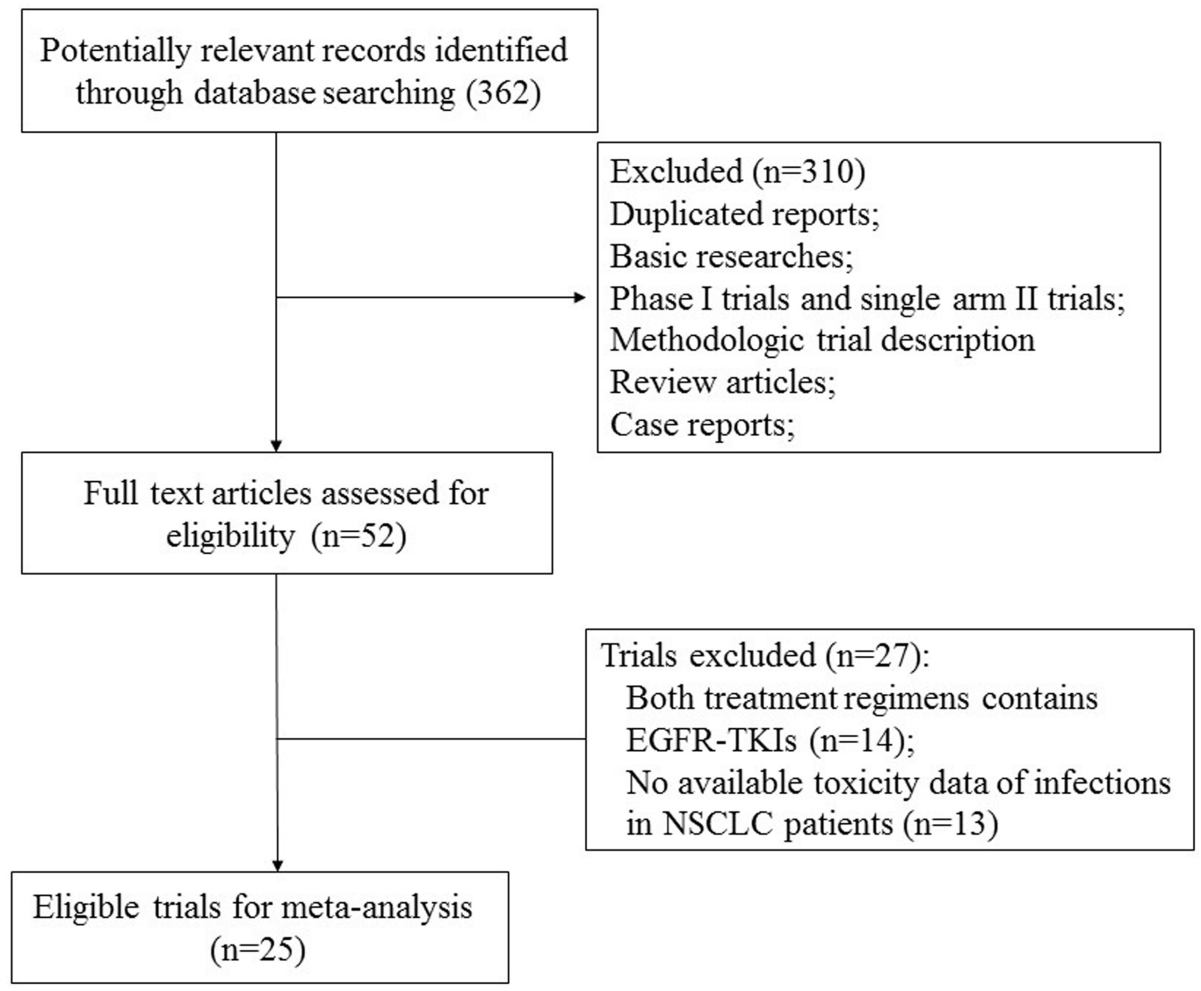
Studies eligible for inclusion in the meta-analysis

We identified a total of 362 related studies through the database search, and retrieved 52 full-text studies for evaluation. The reasons for study exclusion were illustrated in Figure 1. As a result, 25 RCTs with 13,436 patients were included for the present study [17–41]. The baseline characteristics of each trial were presented in Table 1. For the indications of the included studies, there were eight trials in first-line settings, two trials in adjuvant settings, and fifteen trials in the subsequent lines of treatment (maintenance or second line, Table 1). An open assessment of the included trials was carried out by using Jadad scale, and fourteen trials were placebo-controlled, double-blinded randomized trials with Jadad score of 5, and eleven trials had Jadad scores of 3.

**Table 1:** baseline characteristics of 25 trials Included in the Meta-analysis (*n*=17,420)

Studies	Treatment strategy	Enrolled patients (n)	Treatment arms	Median age (years)	Median EGFR-TKIs duration (months)	Median PFS/TTP (months)	Median OS (months)	Patients for analysis	Severe infections	Reported infectious events
Herbst R.S. et al 2004 (INTACT-2)	First-line	1037	Gefitinib 500mg/d plus PC	62	99 days	4.6	8.7	342	NR	Pneumonia, sepsis
			Gefitinib 250mg/d plus PC	61	129 days	5.3	9.8	342	NR	
			Placebo plus PC	63	138 days	5.0	9.9	341	NR	
Giaccone G. et al 2004 (INTACT-1)	First-line	1093	Gefitinib 500mg/d plus GD	61	97d	5.5	9.9	358	NR	Pneumonia
			Gefitinib 250mg/d plus GD	59	150d	5.8	9.9	362	NR	
			Placebo plus GD	61	159d	6.0	10.9	355	NR	
Herbst R.S. et al 2005 (TRIBUTE)	First-line	1059	Erlotinib 150mg/d plus PC	62.7	4.6m	5.1	10.6	526	15	Febrile neutropenia, Pneumonias, sepsis, septic shock
			Placebo plus PC	62.6	5.3m	4.9	10.5	533	7	
Shepherd F.A. et al 2005	Salvage treatment	731	Erlotinib 150mg/d	62	NR	2.2	6.7	485	2	Infection, pneumonitis
			Placebo	59	NR	1.8	4.7	242	5	
Thatcher N. et al 2007	Salvage treatment	1692	Gefitinib 250mg plus BSC	62	2.9	3.0	5.6	1126	30	Pneumonia
			Placebo 250mg plus BSC	61	2.7	2.6	5.1	562	15	
Galzemeier U. et al 2007	First-line	1172	Erlotinib 150mg/d plus GD	60.0	NR	23.7 weeks	43 weeks	579	NR	Neutropenia/febrile neutropenia/neutropenic sepsis
			Placebo plus GD	59.1	NR	24.6 weeks	44.1 weeks	580	NR	
Kelly K. et al 2008 (SWOG S0023)	Maintenance	243	Gefitinib 250mg/d	62	NR	8.3	23	118	3	Pneumonitis
			Placebo	61	NR	11.7	35	125	0	
Kim E.S. et al 2008 (INTEREST)	Second-line	1433	Gefitinib 250mg/d	61	4.4	2.2	7.6	729	23	Lung infections
			Docetaxel	60	3.0	2.7	8.0	715	25	
Cappuzzo F. et al/2010 (SATURN:BO18192)	Maintenance	1949	Erlotinib 150mg qd po	60	NR	12.3weeks	12	433	4	Infections
			Placebo	60	NR	11.1weeks	11	445	0	
Lee D.H. et al 2010 (ISTANA)	Second-line	161	Gefitinib 250 mg/d	57	NR	3.3	NR	81	NR	Pneumonia, septic shock
			Docetaxel	58	NR	3.4	NR	76	NR	
Maemondo M. et al 2010	First-line	230	Gefitinib 250mg/d	63.9	308 days	10.8	30.5	114	3	Pneumonia
			PC	62.6	84 days	5.4	23.6	114	0	
Gaafar R.M. et al/2011 (EORTC 08021)	Maintenance	173	Gefitinib 250mg/d	61	115d	4.1	10.9	85	1	Infections
			Placebo	62	85d	2.3	9.4	86	1	
Natale R.B. et al 2011	Second-line	1240	Erlotinib 150 mg/d	61	8.6 weeks	2.0	7.8	614	NR	Pneumonia, respiratory tract infection
			Vandetanib 300mg/d	61	9.1 weeks	2.6	6.9	623	NR	
Zhou C. et al 2011 (OPTIMAL)	First-line	165	Erlotinib 150mg/d	57	55.5 weeks	13.1	NR	83	1	Infection
			Gemcitabine plus carboplatin	59	10.4 weeks	4.6	NR	72	0	
Ciuleanu T. et al 2012 (TITAN)	Second-line	424	Erlotinib 150mg/d	59	NR	6.3 weeks	5.3	196	1	Infections
			Chemotherapy	59	NR	8.6 weeks	5.5	213	1	
Lee S.M. et al 2012 (TOPICAL)	First-line	670	Erlotinib 150mg/d	77	NR	2.8	3.7	334	5	Pneumonia
			Placebo	77	NR	2.6	3.6	313	1	
Perol M. et al. 2012	Maintenance therapy	464	Observation	59.8	10.9 weeks	1.9	10.8	155	0	Infections
			Gemcitabine	57.9	12 weeks	3.8	15.2	154	2	
			Erlotinib 150mg/d	56.4	12.1 weeks	2.9	11.4	155	4	
Rosell R. et al 2012 (EURTAC)	First-line	174	Erlotinib 150mg/d	65	8.2	9.7	19.3	84	1	Pneumonitis
			Chemotherapy	65	2.8	5.2	19.5	82	1	
Sun J.M. et al 2012 (KCSG-LU08-01)	Second-line		Gefitinib 250mg/d	58	NR	9.0	22.2	68	1	Infections
			Pemetrexed	64	NR	3.0	18.9	67	2	
Goss G.D. et al/2013 (NCIC CTG BR 19)	Adjuvant	503	Gefitinib 150mg/d	66	NR	4.2y	5.1y	251	7	Infection, pneumonitis
			Placebo	67	NR	NR	NR	252	3	
Johson B.E. et al 2013 (ATLAS)	Maintenance	1145	Erlotinib 150mg/d+ bevacizumab	64	72d	4.76	14.39	368	17	Infection
			Placebo +bevacizumab	64	64d	3.71	13.31	367	18	
Kawaguchi T. et al 2014 (DELTA)	Second-line	301	Erlotinib	68	NR	2.0	14.8	150	2	Pneumonitis
			Docetaxel	67	NR	3.2	12.2	150	3	
Li N. et al 2014	Second-line	123	Erlotinib 150mg/d	54.3	NR	4.1	11.7	61	0	Infection
			Pemetrexed	55.1	NR	3.9	13.4	62	0	
Kelly. K. et al 2015 (RADIANT)	Adjuvant	973	Erlotinib 150mg/d	62	NR	46.4	NR	611	8	Pneumonia
			Placebo	61	NR	28.5	NR	343	2	
Soria J.C. et al 2015 (IMPRESS)	Second-line	265	Gefitinib 150mg/d+ Pemetrexed +cisplatin	60	152.5d	5.4	14.8	133	NR	Pneumonia
			Placebo+ Pemetrexed +cisplatin	58	161.5d	5.4	17.2	132	NR	

Abbreviation: TXT, docetaxel; NA, not reported; PC, paclitaxel plus carboplatin; GP, gemcitabine plus cisplatin; BSC, best support care; GD, gemcitabine plus cisplatin; NR, not reported;

### Overall incidence of infections

For the all-grade infectious incidence, a total of 6,593 patients were included for analysis. The pooled incidence was 7.0% (95%CI: 4.7-10.3%). For high-grade infections, a total of 5,977 patients were included for analysis yielding a pooled incidence 2.1% (95%CI: 1.7-2.8%). Additionally, 4,077 patients were included for fatal infections analysis. There was a total of 18 fatal infections reported yielding a pooled incidence of 0.7% (95%CI: 0.4% to 1.0%).

### Peto Odds ratio of infections

**Figure 2:**
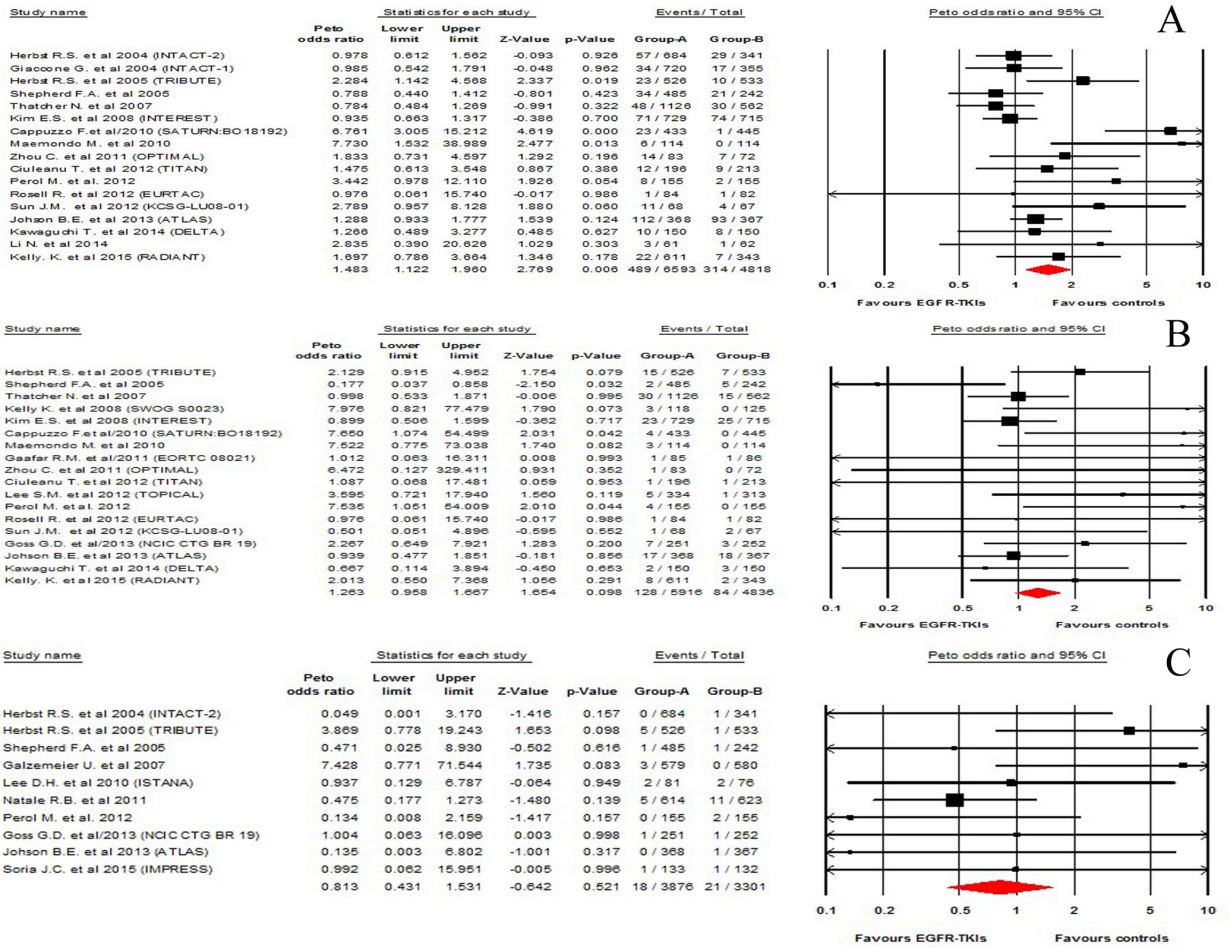
Risk of infections associated with EGFR-TKIs treatment compared with placebo treatment A. all-grade infections, B. high-grade infections, C. fatal infections.

**Figure 3:**
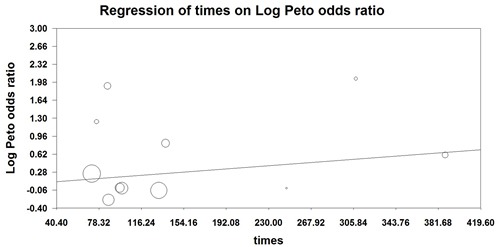
Meta-regression analysis of trends between treatment duration and relative risk of infections: symbols: each study is represented by a circle the diameter of which is proportional to its statistical weight

In order to determine the specific contribution of EGFR-TKIs to the development of infections, a meta-analysis of the Peto OR of infections was performed. Our results showed that the Peto OR of all-grade infections was 1.48 (95%CI: 1.12-1.96, p = 0.006, Figure 2A), while the Peto OR of high-grade infections was 1.26 (95%CI: 0.96-1.67, p = 0.098, Figure 2B). Thus, the use of EGFR-TKIs in NSCLC patients had an increased risk of all-grade infections, but not for high-grade infections. Severe infections could be potentially life-threatening adverse events. There were 18 fatal infections events occurred in the EGFR-TKIs and 21 fatal infections events occurred in control arms, yielding a Peto OR 0.81 (95%CI: 0.43-1.53, p = 0.52, Figure 2C). No significant heterogeneity was found during the Peto OR analysis (Q = 6.64; P = 0.88; I2 = 0%). In addition, we conducted sub-group analysis based on treatment regimens, and demonstrated that the addition of EGFR-TKIs to chemotherapy had a tendency to increase the risk of infections in comparison with chemotherapy alone (Peto OR 1.24, 95%CI: 0.75-3.05, p = 0.39). Similarly, the use of EGFR-TKIs alone had a tendency to increase the risk of all-grade infections when compared to placebo (Peto OR 2.24, 95%CI: 0.27-18.53, p = 0.45) or chemotherapy alone (Peto OR 1.93, 95%CI: 0.88-4.26, p = 0.10). Finally, we carried out a meta-regression analysis to investigate the association between Peto OR of all-grade infections and the length of EGFR-TKIs treatment. The results showed that the longer EGFR-TKIs treatment, and the higher risk of infections with EGFR-TKIs, but this relationship was not statistically significant (P = 0.26, Figure 3).

### Risk of specific infections

We performed analysis to analyze the risk of all-grade infections based on specific type of infection. There was an increased risk of developing EGFR-TKIs-related infections (Peto OR 1.34, 95%CI: 1.08-1.66, p = 0.008) and febrile neutropenia (Peto OR 2.48, 95%CI: 1.31-4.69, p = 0.005), but not for pneumonia (Peto OR 0.97, 95%CI: 0.73-1.29, p = 0.82).

### Publication bias

Egger’s test and Begg’s test was used to detect publication bias. There was no evidence of publication bias for the primary endpoint of this meta-analysis (Peto OR of all-grade infections; Begg’s test p = 0.12; Egger’s test p = 0.06).

## DISCUSSION

The introduction of novel targeted agents into the treatment of cancer has led to improve overall survival of many solid tumors. However, infection is an emerging complication with these drugs, and concerns have arisen regarding the potential risk of developing infections associated with targeted agents. Rafailidis et al [42] conducted the first systematic review in 2007 and demonstrated that there was an increased risk of developing monoclonal antibodies related severe infections but not for fatal adverse events. In consistent with previous results, two later meta-analyses also showed that EGFR-monoclonal antibodies significantly increased the risk of developing severe infections but not for fatal adverse events [16, 43]. Recently, Qi et al. performed another meta-analysis and showed that there was an increased risk of developing all-grade (RR 1.45, p < 0.001) and high-grade (RR 1.59, p < 0.001) infectious events in cancer patients treated with bevacizumab [44]. However, whether the use of EGFR-TKIs would increase the risk of infections in NSCLC remains undetermined.

A total of 17,420 NSCLC patients from 25 RCTs is included for analysis. As far as we known, our study is the first large meta-analysis to show a significantly increased risk of developing EGFR-TKIs related infection (Peto OR 1.48, p = 0.006) in NSCLC patients, but not for high-grade and fatal infectious events. Moreover, we perform a meta-regression analysis to assess the relationship between EGFR-TKIs treatment duration and risk of infections. The result shows that the peto OR of all-grade infections tends to be increased with EGFR-TKIs treatment duration, but it is not statistically significant (p = 0.26). As a result, clinicians should pay more attention to the risk of infections during the administration of EGFR-TKIs. Moreover, clinicians should treat NSCLC patients with any active infection before the initiation of EGFR-TKIs treatment.

Multiple mechanisms might involve in the development of infection. Basic research conducted by Lewkowicz et al [45] found TNF-α induced respiratory burst and phagocytic activity could be enhances by EGFR and its signal pathway. A recent study conducted by Li et al also demonstrated that EGFR play a critical role in the process of Tamm-Horsfall glycoprotein-enhanced neutrophil phagocytosis, and this effect could be suppressed by EGFR inhibitor [46]. However, more high-quality research are still recommended to determine the mechanisms of EGFR-TKIs associated infections.

Several limitations need to be mentioned in the present study. Firstly, as our study is a retrospective analysis of published studies, the baseline characteristics of each studies, such as dosage of EGFR-TKIs, periods of study conduct, and treatment regimens, might be potentially different, which might increase the heterogeneity among included studies. Second, we could not get individual patient data from each published studies, thus we could not perform a comprehensive analysis by adjusting baseline factors that existed between included trials.

## CONCLUSIONS

In conclusion, our study has demonstrated that treatment with EGFR-TKIs in advanced NSCLC is associated with an increased risk of all-grade infections, but not for high-grade and fatal infections. Clinicians should be aware of these risks and provide regular follow-up for these toxicities.

## MATERIALS AND METHODS

### Data sources

We performed this systematic review adhering to the Preferred Reporting Items for Systematic Reviews and Meta-Analyses (PRISMA) statements [47]. Our study was a meta-analysis of published data, and all of these included trials had been approved by the ethics committee, thus the ethical approval in our study was waved. To identify studies for inclusion in this study, we did a broad search of four databases, including Embase, Medline, the Cochrane Central Register of Controlled Trials, and the Cochrane Database of Systematic Reviews, from the date of inception of every database to December 2015. Key words were “erlotinib”, “gefitinib”, “non-small-cell lung cancer”, “lung carcinoma”, “lung neoplasm”, “randomized controlled trial” and “infections”. The search was limited to prospective randomized clinical trials published in English. Each publication was reviewed and in cases of duplicate publications only the most complete, recent, and updated report of the clinical trial was included in the meta-analysis.

### Study selection

To be included for analysis in our systematic review and meta-analysis, the trials had to meet all the following criteria: 1) patients with pathologically confirmed non-small-cell lung cancer; 2) trials comparing therapy with or without EGFR-TKIs (erlotinib and gefitinib); 3) the included study had sufficient data for extraction. We assessed the quality of reports of clinical trials by using the 5-item Jadad scale including randomization, double-blinding, and withdrawals as previously described [48, 49].

### Data extraction and clinical end point

Two independent investigators reviewed the titles and abstracts of potentially relevant studies. We retrieved the full text of relevant studies for further review by the same two reviewers. A third senior investigator resolved any discrepancies between reviewers. If reviewers suspected an overlap of cohorts in a report, they contacted the corresponding author for clarification; we excluded studies with a clear overlap. We extracted the following data: first author’s name, year of publication, number of enrolled subjects, treatment regimens, number of patients in treatment and controlled groups, median age, median treatment duration, median progression-free survival, median overall survival and adverse outcomes of interest (infections). The following adverse outcomes were considered as infectious events and were included in the analyses: Infections (not specified), febrile neutropenia, sepsis, septic shock, lung infection, respiratory tract infection and pneumonia. Adverse events of severe infections (≥grade 3), as assessed and recorded according to the National Cancer Institute’s common terminology criteria (NCI-CTC, version 2 or 3; http://ctep.cancer.gov), were extracted for analysis, which has been widely used in cancer clinical trials.

### Statistical analysis

Statistical analysis of the overall incidence and relative risk for all-grade, high-grade and fatal infections was calculated using comprehensive meta-analysis software version 2.0(Biostat, Englewood, NJ, USA). We used the Peto method to calculate ORs and 95% CIs because this method provided the best confidence interval coverage and was more powerful and relatively less biased than the fixed or random effects analysis when dealing with low event rates [50]. To calculate peto odds ratio (OR), patients assigned to EGFR-TKIs were compared only with those assigned to control treatment in the same trial. Additionally, to test whether effect sizes were moderated by differences in length of treatment, we carried out meta-regressions with difference in median length of experimental treatments (expressed in days) as predictor and relative risk as dependent variable. Between-study heterogeneity was estimated using the χ2-based Q statistic [51]. Heterogeneity was considered statistically significant when P heterogeneity < 0.1. If heterogeneity existed, data was analyzed using a random effects model according to the method of DerSimonian and Laird [52]. In the absence of heterogeneity, the pooled estimate calculated on the basis of the fixed-effects model was reported using an inverse variance method. A statistical test with a p-value less than 0.05 was considered significant. The presence of publication bias was evaluated by using the Begg and Egger tests [53].
